# Measuring low-value care in hospital discharge records: evidence from China

**DOI:** 10.1016/j.lanwpc.2023.100887

**Published:** 2023-08-31

**Authors:** Tianjiao Lan, Lingwei Chen, Yifan Hu, Jianjian Wang, Kun Tan, Jay Pan

**Affiliations:** a HEOA Group, West China School of Public Health and West China Fourth Hospital, Sichuan University, Chengdu, China; b Institute for Healthy Cities and West China Research Center for Rural Health Development, Sichuan University, Chengdu, China; c Ningbo Municipal Center for Disease Control and Prevention, Ningbo, China; d Health Information Center of Sichuan Province, Chengdu, China; e School of Public Administration, Sichuan University, Chengdu, China

**Keywords:** Low-value care, Low-value surgical procedure, Unnecessary cost, Overuse, China

## Abstract

**Background:**

Plenty of efforts have been made to reduce the use of low-value care (the care that is not expected to provide net benefits for patients) across the world, but measures of low-value care have not been developed in China. This study aims to develop hospital discharge records-based measures of low-value surgical procedures, evaluate their annual use and associated expenditure, and analyze the practice patterns by characterizing its temporal trends and correlations across rates of different low-value procedures within hospitals.

**Methods:**

Informed by evidence-based lists including Choosing Wisely, we developed 11 measures of low-value surgical procedures. We evaluated the count and proportion of low-value episodes, as well as the proportion of expenditure and medical insurance payouts for these episodes, using hospital discharge records in Sichuan Province, China during a period of 2016–2022. We compared the count and expenditure detected by different versions of these measures, which varied in sensitivity and specificity. We characterized the temporal trends in the rate of low-value surgical procedures and estimated the annual percent change using joint-point regression. Additionally, we calculated the Spearman correlation coefficients between the risk-standardized rates of low-value procedures which were estimated by multilevel models adjusting for case mix across hospitals.

**Findings:**

Low-value episodes detected by more specific versions of measures accounted for 3.25% (range, 0.11%–71.66%), and constituted 6.03% (range, 0.32%–84.63%) and 5.90% (range, 0.33%–82.86%) of overall expenditure and medical insurance payouts, respectively. The three figures accounted for 5.90%, 8.41%, and 8.38% in terms of more sensitive versions of measures. Almost half of the low-value procedures (five out of eleven) experienced an increase in rates during the period of 2016–2022, with four of them increasing over 20% per year. There was no significant correlation across risk-standardized rates of different low-value procedures within hospitals (mean *r* for pairwise, 0.03; CI, −0.02, 0.07).

**Interpretation:**

Despite overall low-value practices detected by the 11 developed measures was modest, certain clinical specialties were plagued by widespread low-value practices which imposed heavy economic burdens for the healthcare system. Given the pervasive and significant upward trends in rates of low-value practices, it has become increasingly urgent to reduce such practices. Interventions in reducing low-value practices in China would be procedure-specific as practice patterns of low-value care varied by procedures and common drivers of low-value practices may not exist.

**Funding:**

The 10.13039/501100001809National Science Foundation of China (72074163), Taikang Yicai Public Health and Epidemic Control Fund, Sichuan Science and Technology Program (2022YFS0052 and 2021YFQ0060), and 10.13039/501100004912Sichuan University (2018hhf-27 and SKSYL201811).

**Research in context**

**Evidence before this study**

We searched the literature to identify articles that reported the measures of low-value care. We searched PubMed, Web of Science, and China National Knowledge Infrastructure (CNKI) from inception to December 31, 2022, with no language restrictions, using combinations of the terms: “low-value∗” or “low value∗” or “overuse”. We manually selected relevant articles by reading the abstracts. We found Low-value care measures have been well established based on routinely collected administrative datasets in developed countries, and numerous attempts have been made to decrease the utilization of low-value care. There is currently a lack of measures of low-value care in China.

**Added value of this study**

Our study is the first to provide measures of low-value care in China. Based on hospital discharge records from Sichuan Province, China, the study developed 11 measures of low-value surgical procedures. Despite overall low-value practices detected by the 11 developed measures was modest, certain clinical specialties were plagued by widespread low-value practices which imposed heavy economic burdens for the healthcare system. Almost half of the low-value procedures experienced an increase in rates during the period of 2016–2022, with four of them increasing over 20% per year. There was no significant correlation across risk-standardized rates of different low-value procedures within hospitals.

**Implications of all the available evidence**

The developed measures of low-value care provide a straightforward way to assess the extent of specific inappropriate healthcare services, thus informing policymakers and healthcare providers to identify areas where improvements can be made to reduce waste and improve patient outcomes. Given the pervasive and significant upward trends in rates of low-value practices, it has become increasingly urgent to reduce such practices. Interventions in reducing low-value practices in China would be procedure-specific as practice patterns of low-value care varied by procedures and common drivers of low-value practices may not exist.

## Introduction

Low-value care, which is defined as the care that is not expected to provide net benefits for patients, induces unnecessary costs and threatens patients’ safety.^1^ In the past decade, several initiatives, such as Choosing Wisely Campaign enrolling more than ten countries,^2^ have been devoted to the development of evidence-based low-value care lists. In order to measure low-value care, many countries, including the United States,^3^ Australia,^4^ and Canada,^5^ have developed measures (i.e., algorithms) to translate recommendations in such lists into metrics that can be applied to routinely collected administrative data like insurance claims. China, however, has not yet developed such measures despite its initiative for unnecessary cost reduction and healthcare quality improvement since the latest round of health reforms launched in 2009.^6^

Measures of low-value care provide a straightforward way to assess the extent of specific inappropriate healthcare services, thus informing policymakers and healthcare providers to identify areas where improvements can be made to reduce waste and improve patient outcomes. On the other hand, indirect methods of measuring healthcare efficiency, such as estimating total excessive expenditure (or say unnecessary costs) by comparing risk-adjusted expenditure per patient across geographical areas,^7^ could be misleading and challenging for policymakers to act on because unnecessary costs attributable to specific inappropriate services are not determined. Furthermore, such relative measures could fail to capture the true extent of low-value services if their prevalence is consistently high across all providers. In contrast, measures of low-value care can be used to characterize the frequency of low-value care even among the best-performing providers. For developing countries like China, the health expenditure per capita in 2020 was 583 US dollars, which is only half of the global average (1177 US dollars) and one-tenth of that in high-income countries (6180 US dollars).^8^ The figures underscore the severe constraints on healthcare resources in China, highlighting the critical importance of developing measures of low-value care in the country. By limiting specific inappropriate services, even among the best-performing providers, healthcare resources can be reallocated to high-value care, thereby improving the efficiency of the healthcare system in these developing countries.

Based on evidence-based lists and peer-reviewed literature, we developed algorithms to measure low-value surgical procedures. These algorithms are able to be applied to routinely collected administrative hospital discharge records in China. We employed hospital discharge records from 2016 to 2022 in Sichuan Province, China to assess the annual use of these low-value procedures and their associated expenditure, which were detected using two alternative versions of measure (more specific and more sensitive versions). In addition, we determined the practice pattern by characterizing temporal trends in rates of low-value procedures and examining correlations across risk-standardized rates of different low-value procedures within hospitals. To maintain consistency with prior studies, the term “measures of low-value care” will be consistently employed throughout the text to refer to the algorithms used for the identification of low-value care.

## Methods

### Data sources

We based our empirical analysis on Sichuan, a medium-developed province in Mainland China with 2435 hospitals and more than 83 million residents in 2020.^9^ We analyzed hospital discharge records 2016–2022, which covers a total of more than 123 million inpatient episodes. Each episode began with one patient's hospitalization and ended upon her discharge, an episode is therefore essentially synonymous with a hospitalization. The Health Commission of Sichuan Province provided the administrative dataset that records every inpatient admission to hospitals in Sichuan. The dataset contains hospital identifier, expenditure during the hospitalization, inpatients' demographical (e.g., age, sex, and occupation), and clinical information (procedures performed identified by International Classification of Diseases, 9th Revision, Clinical Modification, Volume 3, ICD-9-CM3, and diagnoses assigned identified by International Classification of Diseases, 10th Revision, ICD-10). Unlike insurance claims, this dataset does not include the prescribing or testing records, which limits our measures to surgical procedures.

### Measures of low-value surgical procedures

We based our measures on procedures that have been recommended as low value by Choosing Wisely US,^10^ Choosing Wisely Canada,^11^ Choosing Wisely Australia,^12^ the National Institute for Health and Care Excellence *do not do* recommendations,^13^ the US Preventive Services Task Force *D* recommendations,^14^ Smarter Medicine in Switzerland,^15^ and peer-reviewed literature.^16^ Two authors, both of whom had received bachelor degree in medicine and were familiar with the administrative dataset, independently reviewed the recommendations according to the following criteria to assess their measurability in the hospital discharge records. Any discrepancies were resolved by consensus.

i. *The recommendations should be relevant to inpatient settings.* The procedures that typically occur in outpatient setting were excluded.
ii. *The recommendations should be identifiable with reliability using variables that are present in the hospital discharge records.* Recommendations related to screening, testing, and prescribing were excluded.
iii. *There should be relevant and specific ICD-9-CM3 codes available for the procedure*s. Procedures without identifiable code like Marshall Marchetti Krantz procedure were excluded.

For each selected low-value surgical procedure, we developed an operational definition that is expressed in terms of the following metrics captured in the hospital discharge records: age, sex, ICD-9-CM3, and ICD-10. It should be noted that the value of healthcare frequently varies depending on the clinical context in which services are delivered, and administrative data may not capture all relevant situations due to limited metrics. As a result, our measures of low-value procedures would encounter a tradeoff between sensitivity and specificity.^3^ To address this issue, we adopted the approach utilized by Schwartz et al.^3^ and Brett et al.^17^ to define a narrow definition (more specific, but might exclude some episodes with low-value procedures) and a broad definition (more sensitive, but might include some episodes with appropriate procedures) for procedure as needed. Table 1 presents the operational definitions for 11 measures of low-value surgical procedures applicable to hospital discharge records in China. To assess the adequacy of the narrow and broad definitions in capturing the low-value procedures targeted by the recommendation, we invited five physicians, one from each of the following specialties: gynecology, pediatrics, orthopedics, urology, and gastroenterology. In addition, we invited a health information manager to review whether the listed ICD-9-CM3 and ICD-10 codes were adequate and accurate enough to capture the relevant procedures and diagnoses. See Table S1 in the Supplementary Material for codes used for each low-value care. The complete steps for developing measures of low-value surgical procedures, along with the justification of their operational definitions, can be found in Text S1 of the Supplementary.

**Table 1:** Measures of low-value surgical procedures.^a^

Surgical procedures	Source and supported literature	Qualified episodes definition	Low-value episodes definition	
			Narrower definition (more specific, less sensitive)	Broader definition (less specific, more sensitive)
Hysterectomy	CW^18^	Patients undergoing hysterectomy (including abdominal, vaginal, and laparoscopic approaches), no mention of malignant tumors of female reproductive organs. Minimum age: 18. Sex: female.	Patients undergoing abdominal hysterectomy, no mention of malignant tumors of female reproductive organs, endometriosis, or female pelvic peritoneal adhesions. Minimum age: 18. Sex: female.	Patients undergoing abdominal hysterectomy, no mention of malignant tumors of female reproductive organs. Minimum age: 18. Sex: female.
Bariatric surgical procedures	CW^19^	Patients undergoing bariatric surgical procedures (including open and laparoscopic approaches). Minimum age: 18. Sex: both.	Patients undergoing bariatric surgical procedures (only including open approach). Minimum age: 18. Sex: both.	–
Nasolacrimal duct procedure	CW^20^	Patients with a diagnosis of dacryoadenitis, lacrimal duct inflammation, stenosis, dysfunction, or congenital stenosis and stricture of the lacrimal duct. Maximum age: 12 months. Sex: both.	Probing of nasolacrimal duct in patients with a diagnosis of lacrimal gland inflammation, lacrimal duct inflammation, stenosis, dysfunction, or congenital nasolacrimal duct stenosis. Maximum age: 12 months. Sex: both.	–
Spinal fusion	CW^21^^,^^22^; NICE^23^	Patients with a diagnosis of low back pain; no diagnosis of sciatica, spinal abnormalities, or leg pain. Minimum age: 18. Sex: both.	Patients undergoing spinal fusion with a diagnosis of low back pain and no mention of sciatica, spinal abnormalities, or leg pain. Minimum age: 18. Sex: both.	–
Arthroscopic debridement	CW^24^^,^^25^; literature^16^	Patient with a diagnosis of knee osteoarthritis and no diagnosis of a meniscal tear. Minimum age: 18. Sex: both.	Patients undergoing arthroscopic debridement with a diagnosis of knee osteoarthritis and no mention of a meniscal tear. Minimum age: 50. Sex: both.	Patients undergoing arthroscopic debridement with a diagnosis of knee osteoarthritis and no mention of a meniscal tear. Minimum age: 18. Sex: both.
Vertebroplasty or kyphoplasty	Literature^26^, ^27^, ^28^, ^29^	Patients with a diagnosis of osteoporotic vertebral fracture. Minimum age: 18. Sex: both.	Patients undergoing vertebroplasty or kyphoplasty with a diagnosis of osteoporotic vertebral fracture and no mention of bone cancer, myeloma, or hemangioma. Minimum age: 18. Sex: both.	Patients undergoing vertebroplasty or kyphoplasty with a diagnosis of osteoporotic vertebral fracture. Minimum age: 18. Sex: both.
Renal artery angioplasty or stenting	Liter ature^30^^,^^31^	Patients with a diagnosis of renovascular hypertension, atherosclerosis of renal artery, hypertensive kidney disease, or hypertensive heart and kidney disease; no mention of fibromuscular dysplasia or pulmonary oedema. Minimum age: 18. Sex: both.	Patients undergoing renal artery angioplasty or stenting with a diagnosis of renovascular hypertension, atherosclerosis of renal artery, hypertensive kidney disease, or hypertensive heart and kidney disease; no mention of fibromuscular dysplasia or pulmonary oedema. Minimum age: 18. Sex: both.	–
ERCP	Literature^32^^,^^33^	Patients with a diagnosis of the calculus of bile duct or biliary acute pancreatitis; no diagnosis of cholangitis or bile duct obstruction. Minimum age: 18. Sex: both.	Patients undergoing ERCP with a diagnosis of the calculus of the bile duct or acute biliary pancreatitis and no mention of cholangitis or bile duct obstruction. Minimum age: 18. Sex: both. Exclude admissions from the emergency department.	Patients undergoing ERCP with a diagnosis of the calculus of the bile duct or acute biliary pancreatitis and no mention of cholangitis or bile duct obstruction. Minimum age: 18. Sex: both.
Surgical management of vesicoureteral-reflux	NICE^34^	Patients with a diagnosis of vesicoureteral-reflux-associated uropathy. Maximum age: 11. Sex: both.	Patients undergoing repair surgery on the ureter with a diagnosis of vesicoureteral-reflux-associated uropathy. Maximum age: 11. Sex: both.	–
Anterior colporrhaphy	NICE^35^	Patients with a diagnosis of stress urinary incontinence. Minimum age: 18. Sex: female.	Patients undergoing anterior colporrhaphy with a diagnosis of stress urinary incontinence. Minimum age: 18. Sex: female.	–
Pelvic lymphadenectomy	Literature^36^	Patients with a diagnosis of endometrial cancer. Minimum age: 18. Sex: female.	Patients undergoing pelvic lymphadenectomy with a diagnosis of endometrial cancer and no mention of other female reproductive system cancers. Minimum age: 18. Sex: female.	Patients undergoing pelvic lymphadenectomy with a diagnosis of endometrial cancer. Minimum age: 18. Sex: female.

Abbreviations: CW, Choosing Wisely; NICE, National Institute for Health and Care Excellent; SM, Smart Medicine; ERCP, endoscopic retrograde cholangiopancreatography; NA, not applicable.

a Qualified episodes encompass episodes where inpatients are eligible to receive low-value procedures, regardless of whether they ultimately receive them. On the other hand, low-value episodes specifically pertain to cases where patients undergo low-value procedures. For instance, in the case of hysterectomy, the definition of qualified episodes includes patients undergoing all types of hysterectomy, including both low-value procedures (abdominal approach) and high-value procedures (vaginal and laparoscopic approaches). In contrast, the definition of low-value episodes only includes patients who undergo low-value procedures.

### Counts of low-value episodes and associated expenditure

Low-value episodes specifically pertain to cases where patients undergo low-value procedures, which were captured by the definition of low-value episodes in Table 1. To calculate the proportion of low-value episodes, we defined qualified episodes, with the number of these episodes serving as the denominator. Qualified episodes refer to the episodes where patients are eligible to receive the low-value procedures, regardless of whether they ultimately undergo these procedures (see Table 1 for the definition of qualified episodes). This type of definition, as described by Chalmers et al.,^37^ is known as the *patient-indication measure*. By defining the qualified episodes in this manner, we are able to interpret the proportion of low-value episodes as the proportion of low-value medical decisions.

To determine the expenditure associated with low-value care, we used the total expenditure during an episode. While this method may include additional costs that are not directly induced by low-value care, it allows us to measure the cost of physicians' low-value treatment decisions. Patients typically receive a bundle of treatment procedures, including preoperative exams, anesthesia, and postoperative care, and this approach enables us to bundle the total costs associated with a specific treatment decision. Consequently, the costs of low-value care in this study can be interpreted as the costs of low-value treatment decisions, and the proportion of low-value expenditure can be interpreted as the percentage of cost generated by low-value medical decisions.

Due to the COVID-19 pandemic, China has experienced a nationwide lockdown during a period of January 1, 2020–March 31, 2020. As a result, we excluded the data for this period when calculating the annual counts of low-value procedures and their associated expenditures.

### Statistical analysis

To characterize the practice pattern of low-value care, we characterized the temporal trends in the rate of low-value surgical procedures and estimated the average annual percent change (AAPC) using joint-point regression. We also calculated the spearman correlation coefficients between the risk-standardized rates of different low-value procedures which were estimated by multilevel models adjusting for case mix across hospitals. We used narrow definitions to measure low-value procedures to fit the data, as these definitions cover all procedures, unlike broad definitions. As the lockdown could primarily affected patients' healthcare-seeking behavior more than physicians’ practice patterns, we utilized data from 2016 to 2022 for both analyses, without excluding the lockdown period. Additionally, we conducted sensitivity analyses by excluding the data from the lockdown period to assess the robustness of our findings.

During a specific year, the rate of a particular low-value procedure was calculated by dividing the number of low-value episodes by the number of qualified episodes for that procedure. The AAPC for each procedure from 2016 to 2022 was estimated by the logarithmic Poisson joint-point regression.^38^

To estimate the correlations across procedure-specific risk-standardized rates within hospitals, we first developed a two-level (episode-hospital, level 1 and 2) logistic regression for each procedure. The model incorporated the patients' demographical and clinical characteristics, an indicator variable for the year. We then used the methodology developed by the Centers for Medicare & Medicaid Services^39^ to determine risk-standardized rates for each hospital. In particular, we calculated these rates by dividing the total of *predicted* individual rates of low-value care by the total of *expected* individual rates of low-value care for a given hospital and then multiplying this ratio by the unadjusted average rate of low-value care across all hospitals during our study period. The total of *predicted* individual rates of low-value care was obtained by adding up the probability of each episode where patients receiving low-value care, which was predicted using the hospital random effect, patients' covariates, and year fixed effect, while the total of *expected* individual rates was estimated using the patients' covariates, and year fixed effect. By adjusting for differences in case mix between hospitals, risk-standardized rates facilitate meaningful comparisons of low-value care rates over hospitals. Patients' covariates in the two-level logistic regression include patients' demographical variables (age, age squared, sex, ethnic, marital status, and occupation), patients’ clinical variables (Charlson Comorbidity Index, admission source, insurance type, and length of stay). The definition of each covariate is presented in Table S2 in the Supplementary Materials. To calculate the Spearman correlation coefficients between each pair of low-value procedures within hospitals, we then confined our analysis to hospitals where the number of qualified episodes were greater than 0 for both procedures. We ultimately calculated the Spearman correlation coefficients using the selected hospitals. More estimation details can be found in Text S2 in the Supplementary Materials.

The annual percent change was estimated using Joint-Point software version 5.0, developed by the US National Cancer Institution. The multilevel models were developed in R version 4.1.2, using the *lme 4* package.

### Ethics

The study was approved by the Ethics Committee of West China Fourth Hospital and West China School of Public Health, Sichuan University (Grant number: Gwll2022064).

### Role of funding source

The funders had no involvement in the study design, data collection, data analysis, interpretation, or writing.

## Results

We identified a total of 10,831 annual low-value episodes for 11 measures based on narrow definitions of low-value surgical procedures, accounting for 3.25% of the total qualified episodes in Sichuan Province. For the five low-value procedures applicable to broad definitions, the number of low-value episodes accounted for 5.90% of the corresponding qualified episodes. The proportion of low-value episodes among the 11 procedures exhibited significant variation, ranging from 0.11% of spinal fusion to 71.66% of surgical management of vesicoureteral-reflux in terms of narrow definitions.

According to our narrow definitions, low-value episodes accounted for 30.06 million USD in annual expenditure, which is 6.03% of the total expenditure on qualified episodes. Medical insurance payouts for low-value episodes amounted to 23.17 million USD, or 5.90% of the total medical insurance payouts for qualified episodes. When using broad definitions, the two types of costs represented 8.41% of the overall expenditure and 8.38% of the medical insurance expenses. Consistent with the proportion of low-value episodes, the proportion of low-value expenditure and the proportion of low-value medical insurance payouts varied extensively across different procedures. Table 2 provides the number of low-value episodes and associated expenditure captured by each of the 11 measures of low-value surgical procedure.

**Table 2:** Counts of low-value episodes and associated expenditure detected by measures of low-value care.^a^

Surgical procedures	More specific version of measures						More sensitive version of measures					
	Annual count^b^	PLVE%^c^	Annual expenditure, USD (millions)^d^	PLVS%^e^	Annual MIP, USD (millions)	PLVMIP%^f^	Annual count^b^	PLVE%^c^	Annual expenditure, USD (millions)^d^	PLVS%^e^	Annual MIP, USD (millions)	PLVMIP%^f^
Hysterectomy	6558	19.46	11.58	17.23	9.01	17.29	12,031	35.70	21.52	32.02	16.72	32.11
Bariatric surgical procedures	957	45.11	6.38	45.70	4.62	41.88	–	–	–	–	–	–
Nasolacrimal duct procedure	44	25.79	0.02	17.89	0.02	18.36	–	–	–	–	–	–
Spinal fusion	46	0.11	0.36	1.07	0.18	0.70	–	–	–	–	–	–
Arthroscopic debridement	323	0.20	0.70	0.32	0.56	0.33	412	0.25	0.88	0.41	0.71	0.41
Vertebroplasty or kyphoplasty	2332	35.98	9.07	55.01	7.26	54.34	2375	36.65	9.26	56.18	7.42	55.53
Renal artery angioplasty or stenting	141	0.50	0.83	1.55	0.62	1.43	–	–	–	–	–	–
ERCP	226	0.52	0.79	1.14	0.65	1.20	314	0.73	1.14	1.65	0.93	1.72
Surgical management of vesicoureteral-reflux	85	71.66	0.14	84.63	0.11	82.86	–	–	–	–	–	–
Anterior colporrhaphy	90	2.50	0.11	1.96	0.08	1.92	–	–	–	–	–	–
Pelvic lymphadenectomy	29	0.28	0.10	0.44	0.07	0.37	30	0.29	0.10	0.46	0.07	0.39
Total^g^	10,831	3.25	30.06	6.03	23.17	5.90	15,162	5.90	32.9	8.41	25.84	8.38

Abbreviations: USD, the United States Dollar; PLVE, proportion of low-value episodes; PLVS, proportion of low-value spending; MIP, medical insurance payouts; PLVMIP, proportion of low-value medical insurance payouts; ERCP, endoscopic retrograde cholangiopancreatography.

a The figures in the table were calculated using data from 2016 to 2022 with the exclusion of lockdown period in China (January 1–March 31, 2020).

b Count refers to the number of low-value episodes.

c PLVE = low-value episodes/qualified episodes. See Table 1 for the definitions of qualified episodes and low-value episodes.

d Expenditure refers to the total inpatient cost during an episode, including surgery, medicine, and examination fees.

e PLVS = total expenditure of low-value episodes/total expenditure of qualified episodes.

f PLVMIP = total medical insurance payouts made for low-value episodes/total medical insurance payouts made for qualified episodes.

g The total number of more sensitive version of measures was calculated based on the measures available.

**Fig. 1:**
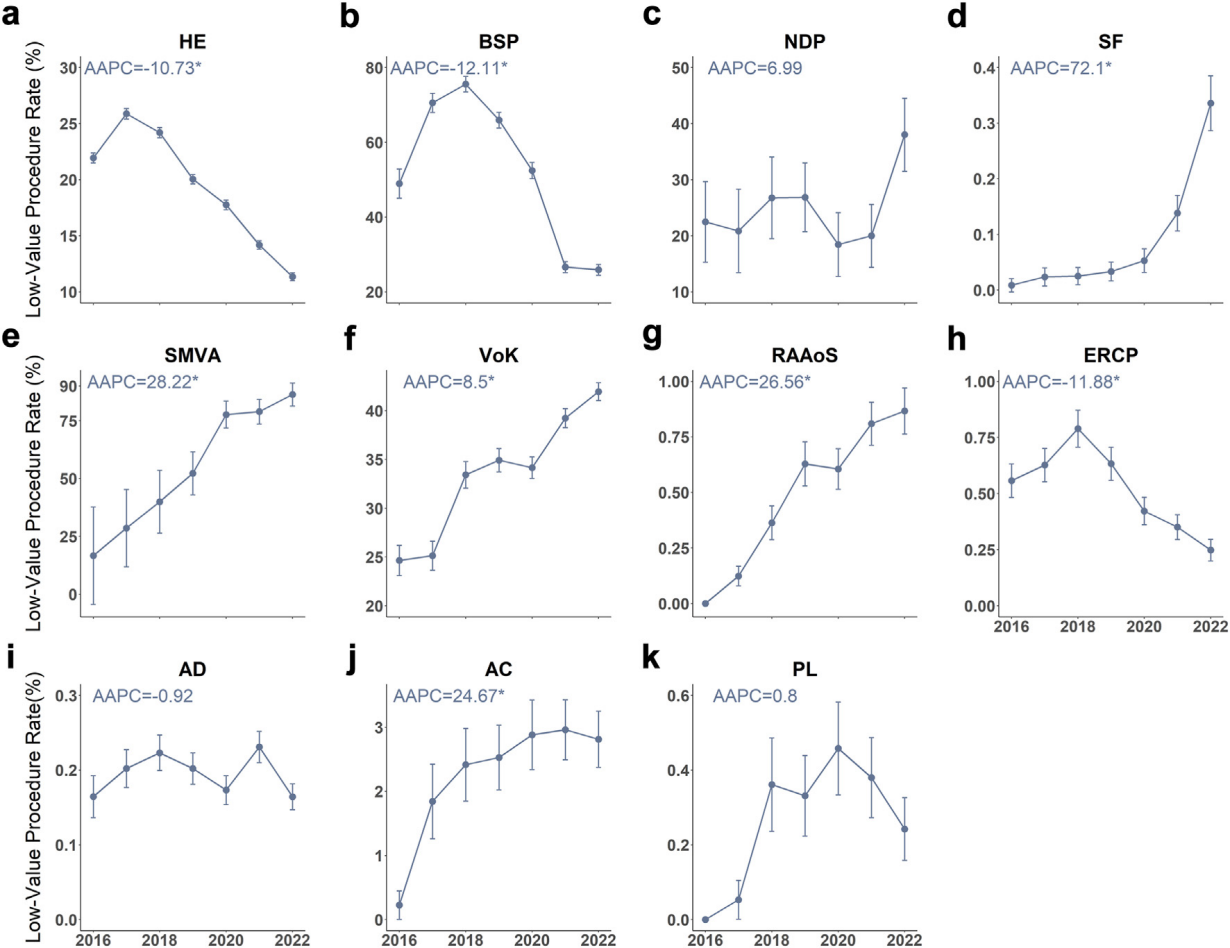
**Temporal trends in the rates of low-value surgical procedures**. Abbreviations: HE, hysterectomy; BSP, bariatric surgical procedures; NDP, nasolacrimal duct procedure; SF, spinal fusion; SMVA, surgical management of vesicoureteral-reflux; AD, arthroscopic debridement; VoK, vertebroplasty or kyphoplasty; RAAoS, renal artery angioplasty or stenting; ERCP, endoscopic retrograde cholangiopancreatography; AC, anterior colporrhaphy; PL, pelvic lymphadenectomy; AAPC, average annual percent change. The dots present the rates of low-value procedures, the vertical bars show their 95% confidence intervals, and the AAPC value marked with an asterisk indicates statistical significance (*P* < 0.05). The rates were calculated by dividing the number of low-value episodes by the number of qualified episodes for each procedure in each year. The average annual percent change was estimated by logarithmic Poisson joint-point regression. The average annual percent change for RAAoS and PL were estimated using data from 2017 to 2022 due to the zero occurrence rates of the two low-value procedures in 2016.

Fig. 1 illustrates the temporal trends in the rates of low-value surgical procedures as detected by our narrow definitions. During the study period, nearly half of low-value procedure rates (five out of eleven) showed an increase in rates, with the *P*-value of AAPC less than 0.05. For four out of the five low-value procedures, the rates increased over 20% per year during 2016–2022, showing significant upward trends. The most dramatic increase in low-value procedure rates was observed in spinal fusion, with a staggering 72.1% annual increase. In comparison, only three procedures exhibited downward trends in low-value procedure rates, with relatively slower rates of change (approximately 10% per year) compared to the increasing procedures. The rates of the remaining three low-value procedures showed no statistically significant change during 2016–2022. The results of annual percent change (APC) for segment periods can be found in Table S3 in the Supplementary Materials. It is worth noting that for the three low-value procedures exhibiting downward trends during 2016–2022, their rates unequivocally started declining from 2018. For example, the rates of low-value endoscopic retrograde cholangiopancreatography increased 20.99% (95% CI, 2.74%–38.86%) per year during 2016–2018, but decreased 24.80% (APC, −24.80% [95% CI, −30.80% to −21.26%]) per year during 2018–2022, respectively.

Table 3 presents the within-hospital correlations between risk-standardized rates of different low-value surgical procedures as detected by our narrow definitions. Half of the Spearman correlation coefficients (28 out of 55) were found to be very small, ranging from −0.10 to 0.10. The majority of these pairwise Spearman correlation coefficients (40 out of 55) were not statistically significant. No consistent correlation pattern was observed among the coefficients with statistical significance. For example, the rates of low-value pelvic lymphadenectomy negatively correlated with vertebroplasty/kyphoplasty but positively correlated with renal artery angioplasty or stenting. Overall, the mean of the correlation coefficients was 0.03, which was not statistically significant (with a confidence interval of −0.02 to 0.07).

**Table 3:** Correlations in hospital use across different types of low-value services.^a^

Surgical procedures	HE	BSP	NDP	SF	AD	VoK	RAAoS	ERCP	SMVA	AC	PL
HE	1										
BSP	0.09	1									
NDP	−0.18	−0.05	1								
SF	**0.10**	−0.01	0.21	1							
AD	**−0.13**	−0.01	−0.12	**0.12**	1						
VoK	−0.07	−0.10	0.01	0.06	**0.29**	1					
RAAoS	−0.02	**−0.20**	0.12	**0.26**	**0.19**	0.05	1				
ERCP	−0.07	−0.04	−0.10	**0.27**	**0.25**	0.05	**0.39**	1			
SMVA	−0.34	0.39	0.33	0.22	−0.07	0.15	0.17	−0.26	1		
AC	0.05	0.08	0.12	−0.07	**−0.10**	−0.01	**−0.14**	**−0.18**	−0.22	1	
PL	0.08	−0.04	−0.1	0.07	−0.07	**−0.19**	**0.10**	0.08	0.04	0.07	1

Abbreviations: HE, hysterectomy; BSP, bariatric surgical procedures; NDP, nasolacrimal duct procedure; SF, spinal fusion; AD, arthroscopic debridement; VoK, vertebroplasty or kyphoplasty; RAAoS, renal artery angioplasty or stenting; ERCP, endoscopic retrograde cholangiopancreatography; SMVA, surgical management of vesicoureteral-reflux; AC, anterior colporrhaphy; PL, pelvic lymphadenectomy.

a Values show Spearman correlation coefficients. The values in bold indicate statistical significance. These coefficients were calculated for each pair of risk-standardized rates of low-value surgical procedures within hospitals. The risk-standardized rates were estimated using two-level logistic regression models which controlled for patient covariates and the indicator of year. See Table S2 for details of covariates and Text S2 for computation details.

Sensitivity analyses conducted on data excluding the lockdown period (January 1 to March 31, 2020) showed remarkably similar results for temporal trends and correlation patterns (Figure S1 and Table S4 in the Supplementary Materials).

## Discussion

In this study, we have developed 11 measures of low-value surgical procedures based on recommendations from evidenced-lists including Choosing Wisely. These measures have been custom-tailored for application in hospital discharge records within the inpatient setting in China. Recognizing the inherent variability in healthcare value across different clinical contexts and the limitations of administrative data in capturing all relevant situations, we established two alternative measures for particular low-value procedures to account for the uncertainty, namely narrow measures (more specific, but might exclude some episodes with low-value procedures) and broad measures (more sensitive, but might include some episodes with appropriate procedures). Using over 123 million episodes from hospital discharge records in Sichuan Province, China, during a period of 2016–2022, we found that the annual use of low-value surgical procedures accounted for a relatively small proportion (3.16% detected by narrow measures and 5.90% by broad measures), and constituted a modest portion of overall expenditure (4.59% and 8.41% by narrow and broad measures) and medical insurance payouts (4.91% and 8.38% by narrow and broad measures). In addition, we found that almost half of the low-value procedures (five out of eleven) experienced an increase in rates during the period of 2016–2022, with four of them increasing over 20% per year. By examining correlations across risk-standardized rates of low-value procedures within hospitals, we found the physicians’ practice patterns of low-value care varied by procedures. 70% of the pairwise correlations showed no statistical significance, and the mean Spearman correlation coefficients was 0.03 (CI, −0.02 to 0.07).

Similar with previous studies,^3^^,^^17^ we based our measures on low-value care lists such as Choosing Wisely. While these lists are primarily from developed countries, we believe that the established recommendations can be generalized to China. The definition of “value” in these lists is based on clinical evidence rather than cost-effectiveness evidence.^40^ For example, only 2% of the recommendations in Choosing Wisely cite cost-effectiveness studies to support their recommendation, compared to 68% of criteria for services that include the words “clinical”, “outcome”, or “harm”.^41^ Since clinical evidence is less dependent on socioeconomic context, we believe it is reasonable to apply these recommendations to China.

Our study, like most studies on measuring low-value care,^3^^,^^5^^,^^42^ relies on administrative database. The primary advantage of the administrative database is its high accessibility, broad coverage, and standardized quantitative metrics, as opposed to (electronic) medical records.^4^ Therefore, the measures we have developed can be readily used to assess low-value care nationwide, thus informing policymakers and healthcare providers to identify areas where improvements can be made to reduce waste and improve patient outcomes. Given that our measures could be directly used to determine unnecessary costs, they are also expected to provide valuable baseline information to guide health policies aimed at reducing waste, such as cost-sharing or diagnosis-related group policies.

Using the developed measures, we evaluated the proportion of low-value surgical procedures, as well as the proportion of low-value expenditure and medical insurance payouts. Physicians typically dominate the process of healthcare delivery due to the information asymmetry between physicians and patients, thus the occurrence of low-value care is heavily influenced by physicians’ treatment preferences (i.e., practice pattern). To capture the proportion of low-value medical decisions induced by physicians and the corresponding expenditure, we employed *patient-indication measure* to define the qualified episodes. Our findings revealed that the proportion of low-value procedures, either assessed using narrow or broad measures, is relatively low. As a result, it constitutes only a modest portion of overall expenditure and medical insurance payouts. However, the proportion varied significantly by procedures, and certain clinical specialties were plagued with widespread low-value practices, which imposed heavy economic burdens for the healthcare system. For example, hysterectomy, a widely performed gynecologic surgery worldwide with a prevalence of more than 1/1000,^43^ is burdened with nearly one-fifth of its procedures being classified as low-value. The significant variation could be attributed to the fact that different physicians demonstrate distinct practice patterns, a phenomenon supported by Schwartz et al.'s findings,^44^ which revealed a notable discrepancy in the provision of low-value care among physicians, even when affiliated with the same hospitals. The determinants influencing the practice pattern of low-value care could be manifold and may differ across various healthcare services; nevertheless, they remain currently unclear.^44^^,^^45^ Attention could be focused on several low-value surgical procedures, including hysterectomy, bariatric surgical procedures, and vertebroplasty/kyphoplasty, due to their high count and associated expenditures, both in absolute figure and proportion.

Comparison with studies that reported prevalence rates of low-value care is limited by several factors, particularly the differences in the low-value services examined as well as the alternative definitions of denominators (i.e., qualified episodes). Most of the studies^46^, ^47^, ^48^ have focused on screening, diagnostic, preventive, or preoperative testing, or imaging, where the prevalence rates are much higher compared to those of low-value surgical procedures in our study. A series of studies from Australia^42^^,^^49^ also assessed surgical procedures, but they used “service denominators” where the number of used services served as denominators, making the comparisons with our study unreasonable. Fortunately, we were able to compare the prevalence rates of three surgical procedures from a national study in the US with our findings. The rates of arthroscopic debridement, vertebroplasty or kyphoplasty, and renal artery angioplasty or stenting in our study are higher than those reported in the US study, where all of the three rates were around zero.^3^ The potential lower prevalence of low-value procedures in the US might potentially be attributed to the higher level of professional competence and the implementation of value-based insurance design^50^ in the country. Much of the evidence concerning low-value surgical procedures has been updated in the last decade. However, in our interviews with physicians, we discovered that many of them have not been monitoring this information, and they are unaware that performing certain surgeries on specific patients may be deemed low value. Additionally, due to the current lack of a detailed value-based insurance design in China, many low-value practices do not receive corresponding penalties, leading to a relatively higher prevalence of low-value services in our study.

Interestingly, during the period of 2016–2022, while half of the low-value procedures experienced an increase in rates, three exhibited downward trends, and their rates unequivocally started declining from 2018. The decrease in the rate of low-value ERCP since 2018 could be attributed to the update of the clinical guideline in 2018,^51^ which provided more detailed indications for ERCP compared to the previous edition. The underlying reasons for the decline in low-value hysterectomy and bariatric surgical procedures, namely those performed via the open approach (low value) instead of the laparoscopic approach (high value), could be more intricate. Considering that the phenomenon was also observed in our previous study,^52^ where we found a significant decrease in the rate of open appendectomy during 2017–2019, we suspect that the decrease could be partly attributed to the rapid diffusion of advanced health technology, such as laparoscopic approach, in China in recent years. With the advancement of health technology, procedures like open hysterectomy for certain patients have been characterized as low value. Hence, getting advanced health technologies into practice in China is a pressing concern. To prevent the potential misuse of novel technologies, it is also crucial to define their appropriate usage scenarios through clinical guidelines throughout the technology diffusion process.

Given that almost half of the low-value procedures showed upward trends, de-implementation of low-value practices is becoming a pressing matter in China. We could ideally estimate the national annual counts of low-value episodes by multiplying our results by 16.8, which is the ratio of the national population to the population in our study area. Based on our eleven developed measures, this suggests that more than 180 thousand low-value episodes occur annually in China, and even a 1% increase would result in thousands of new low-value episodes. Special attention could be directed towards low-value vertebroplasty/kyphoplasty, as their occurrence rate has reached up to 35.98% and is growing at a rate of 8.5% per year.

Analyzing the correlation between the rates of different low-value procedures within hospitals helps reveal the association between practice patterns across procedures. On average, no significant correlation was detected in the study (mean *r* for pairwise, 0.03; CI, −0.02, 0.07). The findings imply that practice patterns of low-value care differ across procedures, as indicated by the significant variation in proportion of low-value procedures. There might be two underlying reasons for the results. First, since different physicians typically have distinct practice patterns,^44^ and our selected procedures involve various clinical specialties, the medical-decision-makers differ, resulting in a lack of correlation between the occurrence rates of these low-value procedures. Second, the practice patterns of the same physician may vary when they encounter patients with different types of illnesses. For example, both hysterectomy and anterior colporrhaphy fall under the field of gynecology, and the two procedures are typically prescribed by the same physician, yet the occurrence rates of low-value procedures show no correlation. As mentioned earlier, the determinants of practice pattern of low-value care are far from clear.^44^^,^^45^ However, recent studies^53^ in the field of implementation science show that educating physicians and the use of medical decision support tools could facilitate the de-implementation of low-value care. Therefore, physicians' expertise may be a crucial factor in shaping the practice patterns of low-value care. On the other hand, as the practice patterns of physicians tend to converge within a medical team or hospital,^54^^,^^55^ intervening at the hospital level to address low-value care could be an effective strategy. Examples of such intervention include the Medicare Pioneer Accountable Care Organization Program in the US.^50^

Some limitations should be noted in this study. Most notably, we only assessed 11 measures of low-value surgical procedures due to the limited clinical information available in hospital discharge records. It is possible that these measures may not be the most relevant to the healthcare system in China. In addition, we were not able to analyze low-value prescribing or testing, even though the large number of recommendations related to these low-value services suggests that they are of great concern. Future studies could base their measures on more informative database, such as insurance claims, to bridge these gaps. Furthermore, despite that we have followed Schwartz et al.^3^ to develop a set of narrow and broad definitions, our measures of low-value procedures may still be susceptible to measurement error due to the lack of gold standards of clinical appropriateness. Last but not least, our empirical analysis was limited to Sichuan Province, and as such, relevant results and subsequent conclusions should be approached with great caution when attempting to generalize them nationwide.

In conclusion, we developed 11 measures for low-value surgical procedures using China's hospital discharge records in this study. Despite overall low-value practices detected by the 11 developed measures was modest, certain clinical specialties were plagued by widespread low-value practices which imposed heavy economic burdens for the Chinese healthcare system. Given the pervasive and significant upward trends in rates of low-value practices, it has become increasingly urgent to reduce such practices. Interventions in reducing low-value practices in China would be procedure-specific as practice patterns of low-value care varied by procedures and common drivers of low-value practices may not exist.

## Contributors

T. Lan and J. Pan conceptualized the study. J. Pan and K. Tan collected the data. T. Lan and L. Chen reviewed the recommendations in the evidence-based lists. T. Lan developed the analysis method. T. Lan implemented the data analysis. T. Lan drafted the manuscript. Y. Hu, J. Wang, K. Tan and J. Pan revised the manuscript. All authors read and approved the final manuscript.

## Data sharing statement

The hospital discharge records that support the findings of this study are available from the Health Commission of Sichuan Province, but restrictions apply to the availability of these data. Data can be made available from the authors upon reasonable request and with permission of the Health Commission of Sichuan Province.

## Declaration of interests

The authors declare that they have no competing interests.
