# Supplementary material for: Measuring low-value care in hospital discharge records: evidence from China
Source: Lancet Reg Health West Pac. 2023 Aug 31;38:100887. doi: 10.1016/j.lanwpc.2023.100887 (PMC10544294; doi:10.1016/j.lanwpc.2023.100887)
Supplement: Supplementary Materials [file mmc1.docx]

**Supplementary Materials**

**Text S1. The complete steps for developing measures of low-value surgical procedures and the justification of their operational definitions.**

Other countries^1–3^ typically follow a two-step approach, a simplified version of process for developing clinical guidelines, to formulating recommendations of low-value care. First, a working group, primarily composed of physicians, is established with the task of proposing initial recommendations for low-value care. The physicians are free to propose any recommendations that are relevant to their respective specialties. However, these initial recommendations should be supported by strong evidence, demonstrating either patient risks or excessive costs outweighing benefits. Subsequently, the Delphi method is employed to facilitate consensus among the working group members on whether the proposed recommendations should be included in the final list. This deliberation process commonly considers two parameters: *a)* the frequency of use and *b)* the relevance to the specific healthcare context of the country.

It is noteworthy that while other countries claim to consider the economic evaluation of tests, treatments, or procedures when developing recommendations, the actual practice often diverges from these claims. A notable example is the Choosing Wisely campaign, which involves over ten countries and aims to develop recommendations on low-value care. Surprisingly, only 2% of the recommendations made in this campaign cite cost-effectiveness studies as supporting evidence for their recommendations.^4^ The reasons for this are unclear, but it is likely due to the limited availability of cost-effectiveness studies compared to clinical efficacy and safety researches.

In this study, we adopted recommendations from other countries after assessing their suitability for implementation in China. Considering the aforementioned processes employed by other countries in developing recommendations, we identified three factors that would impact the suitability of these recommendations in China: *a)* the consideration of economic evaluation studies during the recommendation-making process; *b)* the relevance of the recommendations to China's healthcare system; *c)* whether the identified low-value services are being utilized in China. We consider recommendations based on clinical efficacy and safety to be highly applicable to China, as the evidence supporting them is derived from global randomized controlled trials (RCTs). In fact, our review process revealed significant overlap of many of these recommendations within recommendation lists developed by other countries, including hysterectomy, spinal fusion, and arthroscopic debridement et al. used in our study.

The complete steps for evaluating low-value surgical procedures in this study are as follows. We thoroughly assessed all three factors that could impact the suitability of recommendations in China. The expertise of five physicians and a health information manager we invited validated our operational definitions and codes used for measuring low-value care.

1. Two authors independently reviewed the established list from countries such as the United States, Canada, and others, where numerous studies on measures of low-value care have emerged. The authors, both of whom hold bachelor’s degree in medicine, selected recommendations specifically related to surgical procedures based on the description provided for each recommendation. Any discrepancies were resolved by consensus.
2. For the initially screened recommendations, we further filtered them based on the following criteria: *a)* relevance to China's inpatient settings; *b)* ability to be reliably identified using variables present in hospital discharge records; *c)* availability of relevant and specific ICD-9-CM3 codes for the procedures. The first criterion ensured the recommendation's relevance to the Chinese context, while the second and third criteria ensured their identification using China's hospital discharge records.
3. For the selected recommendations, we reviewed the underlying evidence relied upon by these recommendations. We examined whether economic evaluations were included as a source of evidence and found that none of them incorporated such evaluations. This indicates that these recommendations, which are based on global RCTs, can be applicable in China as well.
4. To facilitate the measure of these recommendations in China's hospital discharge records, we translated the measurable recommendations into operational definitions expressed in terms of the variables in the dataset (Table 1 in the manuscript). In order to ensure the appropriateness and accuracy of the definitions, we sought the evaluation of five clinical physicians, one from each of the following specialties: gynecology, pediatrics, orthopedics, urology, and gastroenterology. The five physicians are all affiliated with third-level hospitals, also known as regional medical centers, in China. Among them, three serve as department heads, and four hold professorship positions. We provided the physicians with the recommendations, supporting evidence and our definitions. We also included the definitions of the same surgical procedures as used in previous literature^5–7^ for their assessment. Subsequently, we made modifications to our definitions based on their feedback.
5. We subsequently conducted a comparison between our final definitions for the same surgical procedures, as used in previous studies,^5–7^ and the definitions employed in those studies. We observed a significant level of consistency between them. This consistency can be attributed to the fact that all relevant recommendations are based on global RCTs, which indicates that the definitions themselves do not vary across different countries. Alongside the expertise of physicians, the observed consistency further contributes to the validation of our definitions.
6. We then assigned ICD-10 and ICD-9-CM3 codes to the disease diagnoses and surgical procedures mentioned in the operational definitions. To validate the adequacy and accuracy of the listed ICD-9-CM3 and ICD-10 codes in capturing the relevant procedures and diagnoses, we enlisted the expertise of a health information manager, who is also affiliated with a third-level hospital in China, for review. We provided the manager with our definitions and codes for her assessment.
7. Using these codes, we measured low-value surgical procedures in the database with the aim of excluding those that did not occur in China. However, the results did not exclude any recommendations. This result also validated all recommendations in our study are relevant to China's healthcare system.

**Text S2. Details on the estimation of correlations across rates of different low-value procedures within hospitals.**

The essential idea of analysis in this part is to estimate the risk-standardized rates for each low-value procedure at each hospital, adjusting for the differences in case mix among hospitals. Similar to the analyses in the last part, we first developed a two-level (episode-hospital, level 1 and 2) logistic regression for each procedure. The model incorporated patients’ demographical and clinical characteristics, hospital random effects and an indicator variable for the year. Thereafter, the Spearman correlation coefficients for each pair of low-value surgical procedures can be calculated by confining our sequent analysis to hospitals where qualified episodes (as defined in Table 1) were greater than 0 for both procedures. The logistic regression model was set as follows.

: Inpatient episode indicator. , where  represents the number of episodes at the hospital .

: Hospital indicator. , where represents the number of hospitals.

Where is the indicator of whether an episode received low-value surgical procedures; represents the *d*-dimensional vector of patients’ covariates including demographical and clinical characteristics; is the hospital random effect; is the 7-dimensional (2016-2022) vector of year indicators, or say year fixed effect.

We then followed the methodology developed by the Centers for Medicare & Medicaid Services^8^ to estimate risk-standardized rates for each hospital *j*. In particular, we calculated these rates by dividing the total of *predicted* individual rates of low-value care by the total of individual *expected* rates of low-value care for a given hospital and then multiplying this ratio by the unadjusted average rate of low-value care across all hospitals during our study period. The approach is analogous to a ratio of *observed* to *expected* used in other types of statistical analyses. We employed the following formula to estimate risk-standardized rates.

*Predicted* individual rates of low-value surgical procedure:

*Expected* individual rates of low-value surgical procedure:

Standard risk ratio:

Risk-standardized rate:

Where represents the number of episodes at the hospital ; is the average low-value care occurrence rate across all hospitals during our study period.

We applied the aforementioned estimator to each low-value surgical procedure *k*. Therefore, we estimated the risk standardized rates for each procedure, namely .

To calculate the Spearman correlation coefficients between each pair of low-value procedures within hospitals, we then confined our analysis to hospitals where qualified episodes (as defined in Table 1) were greater than 0 for both procedures. For the procedure and , the calculation formula is as follows:

Where represents the Spearman Correlation Coefficients calculation function.

**
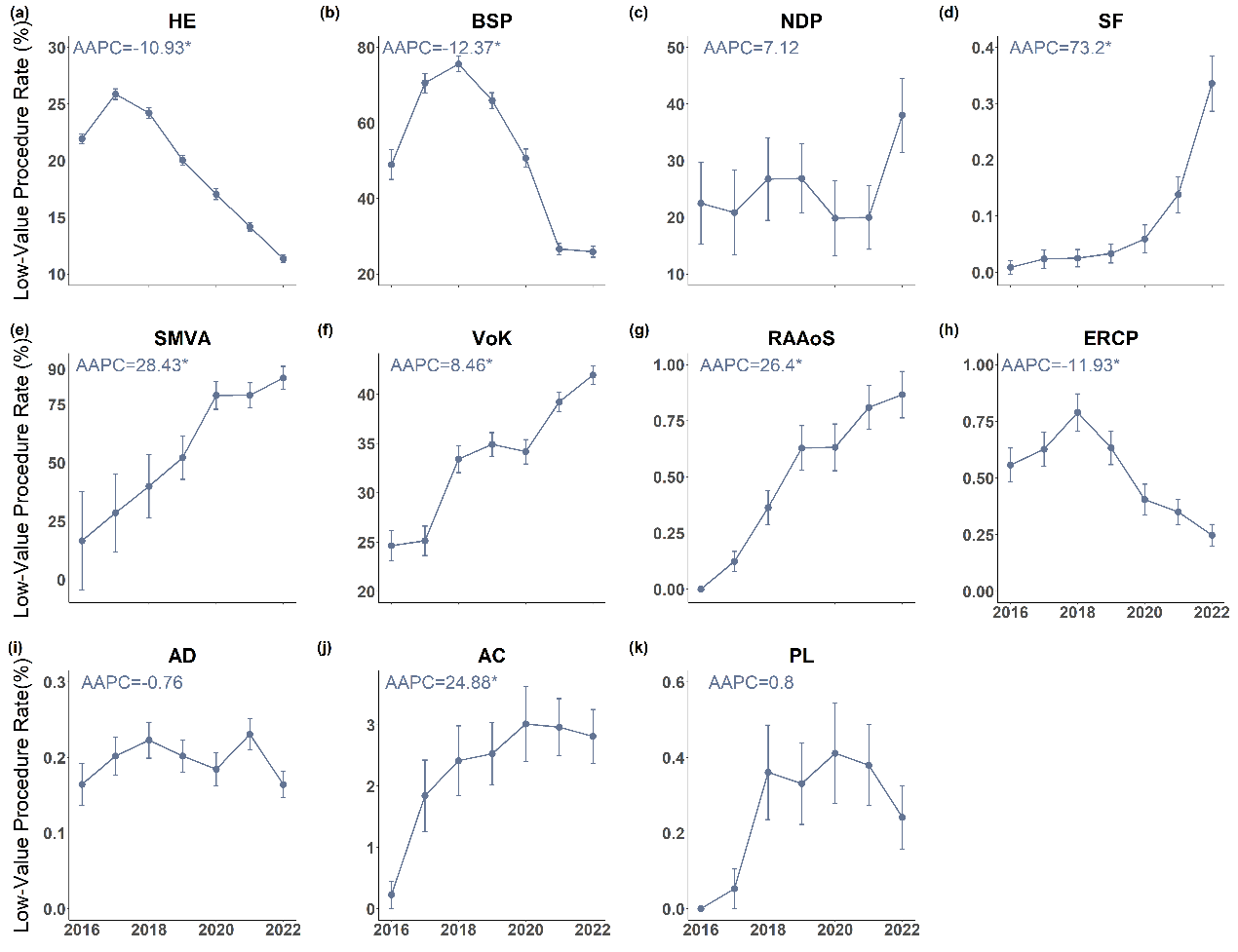
**

**Figure S1. Temporal trends in the rates of low-value surgical procedures using data that excludes the period of lockdown in China.**

Abbreviations: HE, hysterectomy; BSP, bariatric surgical procedures; NDP, nasolacrimal duct procedure; SF, spinal fusion; SMVA, surgical management of vesicoureteral-reflux. AD, arthroscopic debridement; VoK, vertebroplasty or kyphoplasty; RAAoS, renal artery angioplasty or stenting; ERCP, endoscopic retrograde cholangiopancreatography; AC, anterior colporrhaphy; PL, pelvic lymphadenectomy; AAPC, average annual percent change. The dots present the rates of low-value procedures, the vertical bars show their 95% confidence intervals, and the AAPC value marked with an asterisk indicates statistical significance (*P*<0.05). The rates were calculated by dividing the number of low-value episodes by the number of qualified episodes for each procedure in each year. The average annual percent change was estimated by logarithmic Poisson Joint-Point regression. The rates for the year 2020 were calculated using the data from April 1 2020 to December 31, 2020, since the period of lockdown in China was from January 1 to March 31, 2020. The average annual percent change for RAAoS and PL were estimated using data from 2017 to 2022 due to the zero occurrence rates of the two low-value procedures in 2016.

**Table S1. Codes used for measures of low-value surgical procedures.**

| **Measure** | **Codes for qualified episodes** | **Codes for low-value episodes** | |
| --- | --- | --- | --- |
|  |  | **Narrower definition (more specific, less sensitive)** | **Broader definition (less specific, more sensitive)** |
| Hysterectomy | ***Inclusion***:  **ICD-9-CM3**: 68.3-68.6 (hysterectomy).  ***Exclusion***:  **ICD-10**: C51-C58 C79.6 D06-D07 (a malignant tumor of female reproductive organs) | ***Inclusion***:  **ICD-9-CM3**: 68.39 68.49 68.69 (abdominal hysterectomy).  ***Exclusion***:  **ICD-10**: C51-C58 C79.6 D06-D07 (malignant tumor of female reproductive organs); N80 (endometriosis); N73.6 (female pelvic peritoneal adhesions) | ***Inclusion***:  **ICD-9-CM3**: 68.39 68.49 68.69 (abdominal hysterectomy).  ***Exclusion***:  **ICD-10**: C51-C58 C79.6 D06-D07 (a malignant tumor of female reproductive organs) |
| Bariatric surgical procedures | ***Inclusion***:  **ICD-9-CM3**: 43.8903 43.8200 (sleeve gastrectomy); 44.38 44.39 (gastric bypass); 44.68 44.6500 (gastroplasty); 44.95 (gastric banding) 51.33 (biliopancreatic diversion); 45.91 43.6x00-43.6x02 (anastomosis) | ***Inclusion***:  **ICD-9-CM3**: 43.8903 (sleeve gastrectomy open approach); 44.39 (gastric bypass open approach); 44.6500 (gastroplasty open approach); 44.9502 (gastric banding open approach) 51.3300 (biliopancreatic diversion open approach); 45.91 43.6x00 43.6x01 (anastomosis open approach) | - |
| Nasolacrimal duct procedure | ***Inclusion***:  **ICD-10**: H04.0 (dacryoadenitis); H04.3 H04.4 (lacrimal passages inflammation); H04.5 (stenosis and insufficiency of lacrimal passages); Q10.5 (congenital stenosis and stricture of lacrimal duct) | ***Inclusion***:  **ICD-10**: H04.0 (dacryoadenitis); H04.3 H04.4 (lacrimal passages inflammation); H04.5 (stenosis and insufficiency of lacrimal passages); Q10.5 (congenital stenosis and stricture of lacrimal duct)  **ICD-9-CM3**: 09.43(probing of nasolacrimal duct); 09.44 (intubation of nasolacrimal duct) | - |
| Spinal fusion | ***Inclusion***:  **ICD-10**: M54.5 (low back pain)  ***Exclusion***:  **ICD-10**: M54.3 M54.4 (sciatica); S32 M40-M43 (spinal abnormalities); M79.6 (limbs pain) | ***Inclusion***:  **ICD-10**: M54.5 (low back pain)  **ICD-9-CM3**: 81.0 (spinal fusion)  ***Exclusion***:  **ICD-10**: M54.3 M54.4 (sciatica); S32 M40-M43 (spinal abnormalities); M79.6 (limbs pain) | - |
| Arthroscopic debridement | ***Inclusion***:  **ICD-10**: M17 (knee osteoarthritis)  ***Exclusion***:  **ICD-10**: M23 S83.2 M22.4 S83.3 (meniscal tear) | ***Inclusion***:  **ICD-10**: M17 (knee osteoarthritis)  **ICD-9-CM3**: 80.1604 80.26 80.8600x009 80.8602 81.4700x015 (arthroscopic debridement)  ***Exclusion***:  **ICD-10**: M23 S83.2 M22.4 S83.3 (meniscal tear) | |
| Vertebroplasty or kyphoplasty | ***Inclusion***:  **ICD-10**: M80 S22.0 S22.1 S32.0 (osteoporotic vertebral fracture) | ***Inclusion***:  **ICD-10**: M80 S22.0 S22.1 S32.0 (osteoporotic vertebral fracture)  **ICD-9-CM3**: 81.65 (vertebroplasty); 81.66 (kyphoplasty)  ***Exclusion***:  **ICD-10**: C40 C41 C79.5 (bone cancer); C90.0 C90.2 (myeloma); D18.0 (hemangioma) | ***Inclusion***:  **ICD-10**: M80 S22.0 S22.1 S32.0 (osteoporotic vertebral fracture)  **ICD-9-CM3**: 81.65 (vertebroplasty); 81.66 (kyphoplasty) |
| Renal artery angioplasty or stenting | ***Inclusion***:  **ICD-10**: I15.0 (renovascular hypertension); I70.1 (atherosclerosis of renal artery); I12 (hypertensive kidney disease); I13 (hypertensive heart and kidney disease)  ***Exclusion***:  **ICD-10**: Q79.800x007 (fibromuscular dysplasia); J81 (pulmonary oedema) | ***Inclusion***:  **ICD-10**: I15.0 (renovascular hypertension); I70.1 (atherosclerosis of renal artery); I12 (hypertensive kidney disease); I13 (hypertensive heart and kidney disease)  ICD-9-CM3: 39.5900x010 39.5002 (Renal artery angioplasty); 39.9016(Renal artery stenting)  ***Exclusion***:  **ICD-10**: Q79.800x007 (fibromuscular dysplasia); J81 (pulmonary oedema) | - |
| ERCP | ***Inclusion***:  **ICD-10**: K80.4 K80.5 (calculus of bile duct); K85.1 (biliary acute pancreatitis)  ***Exclusion***:  **ICD-10**: K80.3 (calculus of bile duct with cholangitis); K83.0 (cholangitis); K83.1 (obstruction of bile duct) | ***Inclusion***:  **ICD-10**: K80.4 K80.5 (calculus of bile duct); K85.1 (biliary acute pancreatitis)  **ICD-9-CM3**: 51.10 (ERCP)  ***Exclusion***:  **ICD-10**: K80.3 (calculus of bile duct with cholangitis); K83.0 (cholangitis); K83.1 (obstruction of bile duct) | |
| Surgical management of vesicoureteral-reflux | ***Inclusion***:  **ICD-10**: N13.7 (vesicoureteral-reflux) | ***Inclusion***:  **ICD-10**: N13.7 (vesicoureteral-reflux)  **ICD-9-CM3**: 56.7 56.8 (vesicoureteral-reflux-associated repair surgery ^a^) | - |
| Anterior colporrhaphy | ***Inclusion***:  **ICD-10**: N39.3 (stress urinary incontinence) | ***Inclusion***:  **ICD-10**: N39.3 (stress urinary incontinence)  **ICD-9-CM3**: 70.5101 70.5102 70.5400x001 70.5400x002 (anterior colporrhaphy) | - |
| Pelvic lymphadenectomy | ***Inclusion***:  **ICD-10**: C54.1 D07.0 (endometrial cancer) | ***Inclusion***:  **ICD-10**: C54.1 D07.0 (endometrial cancer)  **ICD-9-CM3**: 40.2909 40.5910 40.5912 (pelvic lymphadenectomy)  ***Exclusion***:  **ICD-10**: C51-C58 [except C54.1] C79.6 D06 D07 [except D07.0] C77 C79 (other female reproductive system cancers) | ***Inclusion***:  **ICD-10**: C54.1 D07.0 (endometrial cancer)  **ICD-9-CM3**: 40.2909 40.5910 40.5912 (pelvic lymphadenectomy) |

Abbreviations: ERCP, endoscopic retrograde cholangiopancreatography.

^a^ Surgical procedures that patients with vesicoureteral-reflux had undergone based on our preliminary analysis.

**Table S2. Definitions of covariates^a^**

| **Variables** | **Definition** |
| --- | --- |
| ***Patients’ demographical characteristics*** | |
| Age |  |
| Age squared | The square of age. |
| Sex | A category variable, including female and male |
| Ethnic | A category variable, including Han and ethnic minorities |
| Marital status | A category variable, including married, unmarried (the windowed, the divorced, and the never married), and unknown |
| Occupation | A category variable, including at work (civil servants, clerks, enterprise managers, workers, soldiers, freelancers, and self-employed individuals), retired, and others (including farmers, students, and the unemployed) |
| ***Patient’s clinical characteristics*** | |
| Charlson Comorbidity Index | A continuous variable was used to control for patients' comorbidities, which was calculated according to Charlson et al.'s method.^9^ |
| Admission source | A category variable, including the emergency department within the hospital, the outpatient department within the hospital, transferred from other hospitals, and others. |
| Insurance type | A category variable, including UEBMI, URBMI, NCMS, full self-expense, and others (commercial medical insurance and government-funded expense). |
| Length of stay | A continuous variable was used to control for the severity of the disease, with units measured in days. |

Abbreviations: UEBMI, Urban employment basic medical insurance; URBMI, Urban resident basic medical insurance; NCMS, New cooperative medical scheme.

^a^ To avoid data sparsity in certain categories of categorical variables, we combined some of the raw categories. The categories listed in the table for category variables were used for statistical analysis.

**Table S3. The annual percentage change in low-value procedure rates during 2016-2022 using Joint-Point regression^a^.**

| **Surgical Procedures** | **Segment** | **Period** | **APC (95%CI)** |
| --- | --- | --- | --- |
| Hysterectomy | 1 | 2016-2018 | 4.53 (-5.66, 14.3) |
|  | 2 | 2018-2022 | -17.5 (-24.57, -14.51) |
| Bariatric surgical procedures | 1 | 2016-2018 | 25.08 (-7.88, 92.01) |
|  | 2 | 2018-2022 | -26.32 (-40.99, -19.87) |
| Nasolacrimal duct procedure | 1 | 2016-2020 | -4.34 (-18.09, 11.71) |
|  | 2 | 2020-2022 | 29.70 (-14.37, 96.46) |
| Spinal fusion | 1 | 2016-2020 | 42.20 (-16.5, 74.54) |
|  | 2 | 2020-2022 | 152.09 (120.42, 201.97) |
| Surgical management of vesicoureteral-reflux | 1 | 2016-2020 | 41.18 (37.47, 48.02) |
|  | 2 | 2020-2022 | 5.76 (3.07, 8.43) |
| Vertebroplasty or kyphoplasty | 1 | 2016-2018 | 16.65 (-30.54, 95.90) |
|  | 2 | 2018-2022 | 6.80 (-1.65, 15.98) |
| ERCP | 1 | 2016-2018 | 20.99 (2.74, 38.86) |
|  | 2 | 2018-2022 | -24.80 (-30.80, -21.26) |
| Anterior colporrhaphy | 1 | 2016-2018 | 13.06 (-79.04, 509.71) |
|  | 2 | 2018-2022 | -4.33 (-33.99, 38.66) |
| Arthroscopic debridement | 1 | 2016-2018 | 82.67 (-3.08, 378.67) |
|  | 2 | 2018-2022 | 2.99 (-20.65, 29.03) |

Abbreviations: ERCP, endoscopic retrograde cholangiopancreatography; APC, annual percentage change; CI, confidence interval.

^a^ The APC statistics for the procedures of “renal artery angioplasty or stenting” and “pelvic lymphadenectomy” are not available. This is due to the fact that the occurrence rate of these low-value procedures was zero in 2016, resulting in only six available data points for analysis. It is worth noting that segment APC analysis is not recommended when the number of data points is less than seven (https://surveillance.cancer.gov/help/joinpoint/setting-parameters/method-and-parameters-tab/number-of-joinpoints).

**Table S4. Correlations in risk-standardized rate across different types of low-value surgical procedures using data that excludes the period of lockdown in China^a^.**

| **Surgical**  **Procedures** | **HE** | **BSP** | **NDP** | **SF** | **AD** | **VoK** | **RAAoS** | **ERCP** | **SMVA** | **AC** | **PL** |
| --- | --- | --- | --- | --- | --- | --- | --- | --- | --- | --- | --- |
| HE | 1 |  |  |  |  |  |  |  |  |  |  |
| BSP | 0.08 | 1 |  |  |  |  |  |  |  |  |  |
| NDP | -0.15 | -0.05 | 1 |  |  |  |  |  |  |  |  |
| SF | **0.11** | -0.01 | 0.21 | 1 |  |  |  |  |  |  |  |
| AD | **-0.13** | -0.01 | -0.1 | **0.13** | 1 |  |  |  |  |  |  |
| VoK | -0.08 | -0.11 | -0.01 | 0.05 | **0.28** | 1 |  |  |  |  |  |
| RAAoS | -0.03 | **-0.21** | 0.13 | **0.26** | **0.19** | 0.05 | 1 |  |  |  |  |
| ERCP | -0.07 | -0.05 | -0.09 | **0.27** | **0.24** | 0.05 | **0.38** | 1 |  |  |  |
| SMVA | -0.34 | 0.39 | 0.23 | 0.22 | -0.07 | 0.18 | 0.17 | -0.22 | 1 |  |  |
| AC | 0.04 | 0.07 | 0.05 | -0.07 | **-0.11** | -0.01 | **-0.14** | **-0.18** | -0.22 | 1 |  |
| PL | 0.09 | -0.06 | -0.1 | 0.06 | **-0.09** | **-0.2** | **0.11** | 0.08 | -0.03 | 0.05 | 1 |

Abbreviations: HE, hysterectomy; BSP, bariatric surgical procedures; NDP, nasolacrimal duct procedure; SF, spinal fusion; AD, arthroscopic debridement; VoK, vertebroplasty or kyphoplasty; RAAoS, renal artery angioplasty or stenting; ERCP, endoscopic retrograde cholangiopancreatography; SMVA, surgical management of vesicoureteral-reflux; AC, anterior colporrhaphy; PL, pelvic lymphadenectomy.

^a^ Values show Spearman correlation coefficients. The values in bold indicate statistical significance. These coefficients were calculated for each pair of risk-standardized rates of low-value surgical procedures within hospitals. The risk-standardized rates were estimated using two-level logistic regression models which controlled for patient covariates and the indicator of year. See Table S2 for details of covariates and Text S2 for computation details. The data from January 1 to March 31, 2020 (the period of lockdown in China) was excluded when estimating the risk-standardized rates and the Spearman correlation coefficients.

**eReferences**

1. Choosing Wisely Canada Group. *Medical Professional Society Handbook*.; 2020. Accessed June 24, 2023. https://choosingwiselycanada.org/wp-content/uploads/2022/02/2020-10-01_Medical-professional-society-handbook-V1.4.pdf

2. Choosing Wisely Australia Group. Developing Choosing Wisely Australia recommendations. Accessed June 24, 2023. https://www.choosingwisely.org.au/recommendations

3. Pramesh CS, Chaturvedi H, Reddy VA, et al. Choosing Wisely India: ten low-value or harmful practices that should be avoided in cancer care. *LANCET ONCOLOGY*. 2019;20(4):E218-E223.

4. Pandya A. Adding Cost-effectiveness to Define Low-Value Care. *JAMA-JOURNAL OF THE AMERICAN MEDICAL ASSOCIATION*. 2018;319(19):1977-1978.

5. Schwartz AL, Landon BE, Elshaug AG, Chernew ME, McWilliams JM. Measuring Low-Value Care in Medicare. *JAMA INTERNAL MEDICINE*. 2014;174(7):1067-1076.

6. Badgery-Parker T, Pearson SA, Chalmers K, et al. Low-value care in Australian public hospitals: prevalence and trends over time. *BMJ QUALITY & SAFETY*. 2019;28(3):205-214.

7. Chalmers K, Pearson SA, Badgery-Parker T, Brett J, Scott IA, Elshaug AG. Measuring 21 low-value hospital procedures: claims analysis of Australian private health insurance data (2010-2014). *BMJ OPEN*. 2019;9(3).

8. Yale New Haven Health Services Corporation. *2022 Procedure-Specific Complication Measure Updates and Specifications Report*.; 2022:38-39. Accessed March 27, 2023. https://www.cms.gov/files/document/2022-measure-updates-procedure-specific-complication-measure-updates-and-specifications-report.pdf

9. Charlson ME, Pompei P, Ales KL, MacKenzie CR. A new method of classifying prognostic comorbidity in longitudinal studies: development and validation. *J Chronic Dis*. 1987;40(5):373-383. doi:10.1016/0021-9681(87)90171-8
